# Spinal loop rectangle and sub laminar wiring as a technique for scoliosis correction

**DOI:** 10.4103/0019-5413.58606

**Published:** 2010

**Authors:** Shekhar Y Bhojraj, Raghuprasad G Varma, Abhay M Nene, Sheetal Mohite

**Affiliations:** Consultant Spine Surgeon, Lilavati Hospital and MRC and Breach Candy Hospital, Mumbai, India; 1 Consultant Spine Surgeon, Dr. L H Hiranandani Hospital, Wockhardt Hospital and Fortis Hospital, Mumbai, India; 2 Consultant Spine Surgeon, P D Hinduja National Hospital, Mumbai, India; 3 Consultant Spine Surgeon, Lilavati Hospital and MRC and Shushrusha Hospital, Mumbai, India

**Keywords:** Cost-effective, scoliosis surgery, spinal loop rectangle and sublaminar wires

## Abstract

**Background:**

Most literature popularizes the efficacy of third generation instrumentation in the surgical correction of spinal deformities. A cheap and effective scoliotic deformity correction method is reviewed in this article. The aim of this study is to evaluate the efficacy of spinal loop rectangle and sub laminar wires as a modality for spinal deformity correction and its co-relation with patients' satisfaction and clinical outcome.

**Material and Methods:**

Thirty six patients of scoliotic spinal deformities with various etiologies (congenital-4, idiopathic- 25, neurofibromatosis-3, neuromuscular-2 and ‘syndromic’-3) with ages ranging from 8 to 23 years underwent corrective posterior spinal arthrodesis with stainless steel Hartshill loop rectangle and sublaminar wires. Clinicoradiological evaluation was done at an average follow-up of 6 ½ years (min-2 ½, years). Along with clinicoradiological outcome, patient satisfaction (as per the SRS 24), was accounted.

**Results:**

Average preoperative Cobb's angle were 73.25° in the entire group and 66.48° in the idiopathic group. Average percentage correction was 64.34% in the entire group and the (average degree of correction was 47.13). In the idiopathic group, the respective values were 69.19% and 46°. Loss of correction in the whole group was 2.2° at two year follow up. Sagittal profiles, truncal balance were well corrected too; minimal complications were seen. Patient satisfaction results were encouraging in 36 patients as per – SRS24). About 80.2% patients were ready to undergo the same surgery if required. (SRS24).

**Conclusion:**

Segmental spinal fixation with locally made spinal loop rectangle and sublaminar wires is comparable as a modality to correct scoliotic spinal deformities.

## INTRODUCTION

Lange, (1902), was the first surgeon to wire a rod to the spine in case of spondylitis, it was Resina and Ferreira,^1^ from Portugal, who in 1963 pioneered spine fixation with segmental wiring for the treatment of scoliotics curves. Luque and Cardozo,^2^,^3^ in the later 1970s, popularized the method using sublaminar wires to attach each vertebra to his designed L-shaped rods. Later the wiring of the apical vertebrae to the Harrington rod became a popular modality to correct coronal spinal deformities, In the early 1980s, Dove^4^ delineated the Hartshill rectangle in England, and the Lea-Plaza^5^ frame was designed in Uruguay (1986). All these developments represent the main landmarks in the concept of segmental fixation.

Segmental spinal instrumentation, done by sublaminar wiring and Hartshill loop rectangle, had fallen out of favor in comparison to the third generation implants. The main reasons were: a) fear of long term effects of wires within the spinal canal and high incidence of neurological complications due to the wires,^6^ (though mostly dysesthesias and occasional paraplegias in literature). b) Inadequate corrective forces, especially rotatary control in the instrumented segment was also a deficiency claimed, c) sagittal profile correction and disputed maintenance.

We conduct this study to evaluate the efficacy of spinal loop rectangle and sublaminar wires as a modality for spinal deformity correction and its co-relation with patients' satisfaction and clinical outcome. Focus of the study is predominantly on the achievement of significant deformity correction with cost effective instrumentation and document complications.

## MATERIALS AND METHODS

The study restrospectively evaluated 36 consecutive patients operated for Scoliosis of varying etiologies, using sublaminar wires and spinal rectangle from January 2000 and June 2006. There were five males and 31 females, and the average age of the patients in our study was 14.2 years (range 8 to 23 years). The etiological distribution was as follows: congenital-4, idiopathic 25, neurofibromatosis-3, neuromuscular-2 and ‘syndromic’-3. The idiopathic group were further classified by King – Moe's classification as follows; type 2 (n=10), type 3 (n=9), type 4 (n=6) patients. No patients belonged to type 1 and type 5.

**Figure 1:**
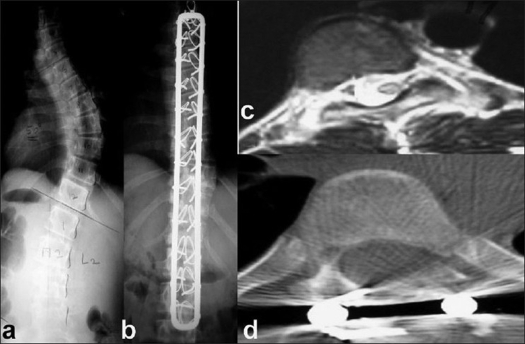
(a) X-ray dorso lumbar spine antero posterior view showing idiopathic scoliotic deformity (b) X-ray dorso lumbar spine antero posterior view showing good coronal correction of an idiopathic scoliotic deformity at five years post operative (c) MRI T2WI Axial shows rotation of the vertebral body (Pre operative) (d) CT Scan Axial image shows appreciable derotation post operatively

The average Cobb's angle in the entire group (n=36) was 73.25°C and in the idiopathic group (n=25) was 66.48°C. Seven patients were Left convex curves, while 29 were right convex curves. The sagittal profile was measured between T2 – T12 for thoracic kyphosis and L1 – S1 for lumbar lordosis. The normal thoracic kyphosis was taken as 20 – 40° and lumbar lordosis as 40 – 60°. In the ‘idiopathic group’ the thoracic curves average 68° and the lumbar curves 48° [Figure 1].

Fulcrum bending films were done in only in the later part of the study group, especially in the idiopathic group and therefore is not included as a criterion in this study.Junctional kyphosis was evaluated in all patients and angle measurements were taken between the lower endplate of the uppermost instrumented vertebra and the upper endplate of the two vertebrae supraadjacent, defining PJK only if values > 10° and at least 10° more than the preoperative measurements.

Preoperative SRS 24 questionnaires were completed by 36 patients, while Cobb's angles and radiological sagittal profiles were measured by an independent observer. Preoperative MRI scans were done in all patients to document Intraspinal anomalies.

Preoperative decision making of an anterior release was made on the basis of pubertal status of the patient (especially in Premenarchal), rigidity of curve (esp. in rigid curves), Rissers grade (especially in Lower Risser grades). We have used the Fulcrum Bending Views in the latter part of our series though it is not included in the present study.

The Rib Hump was evaluated clinicoradiologically. If significant humps were observed Internal costoplasty were done if an anterior release was planned. If only a posterior procedure was planned then the rib humps were corrected by posterior costoplasty. Patients routinely followed up at 3, 6 and 12 months in the first year after surgery, and then once a year.

At every visit, included coronal Cobb's angle (anteroposterior view), Sagittal balance (lateral view) (forward bending shoot through views (for Rib Hump) were measured. CT scans were done at the apex to document derotation in eight patients.

All 36 patients attended a follow-up scoliosis clinic and filled the SRS 24 patients' satisfaction questionnaire and Roland Morris low back pain questionnaire, apart from clinicoradiological evaluation at this time.

### Operative procedure

**Figure 2:**
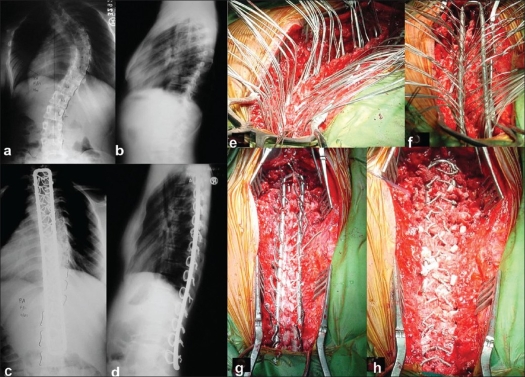
Pre operative (a,b) and post operative (c,d) x-rays of dorso lumbar spine shows good correction, balance and maintenance of a scoliotic deformity at 4 ½ years follow up. Intraoperative photographs show different stages of instrumentation (e-h)

Once the midline posterior exposure was complete lateral up to the transverse processes, the sublaminar spaces are created by cutting the interspinous ligaments and midline ligamentum flavum. This is a key step. Once the sublaminar spaces are exposed in the entire levels to be instrumented 20 gauge stainless steel double looped wires are passed at each level (in caudal to cephalad direction). The facetal preparation for fusion is then done which also helps some amount of posterior release. The size of loop rectangle required is then measured and adequately contoured (very important for sagittal balance). Wires are tied onto the loop rectangle with cephalad wire ends inside the loop rectangle and caudad wire ends outside the loop rectangle at each level (except at the lower end of the construct where it is reversed to prevent the down slide of the construct)/ Tightening of concave wires first at each level was done which causes translation of curve on to the loop rectangle and then the wires at convex sides to play a role in derotating the curve. Two more final tightening, correct the spinal deformity onto the loop rectangle. After packing the sublaminar spaces with gel foam, thorough shingling and graft bed preparation for posterior fusion is undertaken [Figure 2].

## RESULTS

Twenty three patients underwent only posterior surgery and 13 patients underwent single stage anterior release and posterior instrumentation. Posterior costoplasty was done in four patients. Autogenous bone graft from the posterior iliac crest was used in all patients, mixed with hydroxy apatite crystals in 32 patients.

**Figure 3:**
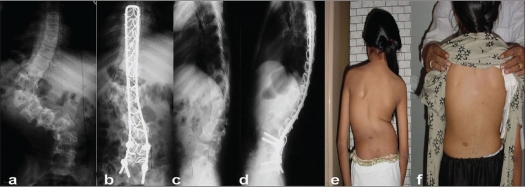
Preoperative and post operative x-rays antero posterior and lateral views (a-d) shows a novel concept of sacral fixation using pedicular screws in L4-S1, fixed to the split arms of the rectangle, in severe dystrophic curve in neurofibromatosis. Preoperative and post operative clinical photographs (e,f) shows significant correction in both planes and good spinal function at five years follow up

In three patients, lower end of the spinal rectangle was incorporated with pedicle screws for a hybrid construct because of double major curves with rigid lumbar curves warranting extending the construct into the sacrum for which wires are inadequate [Figure 3].

We used 20 gauge, double looped, pre-contoured stainless steel sublaminar wires and a 6 mm thick stainless steels spinal rectangle, both manufactured locally, for the spinal instrumentation.

Intra operative monitoring of somatosensory evoked potentials was done in all the cases.

Postoperative bracing was done in the first 20 patients in the series, in the form of a custom made molded whole body jacket, for three months, especially in the congenital and dystrophic (neurofibromatosis) groups (n= 7). Postoperative mobilization was done at an average of eight days (5 to 16 days). The practice of bracing was in our initial group of patients as part of a routine though discontinued as our experience increased, and today we do not advocate routine postop bracing.

Follow-up ranged from 30 to 96 months, the average being 78 months. For the entire group of 36 patients the average scoliosis in the coronal plane (Cobb's angle) measured 73.25° pre-operatively and 26.12° immediate post operatively. In the idiopathic group, the respective values were 66.48°C pre-operative, and 20.4° immediate post operative.

**Figure 5:**
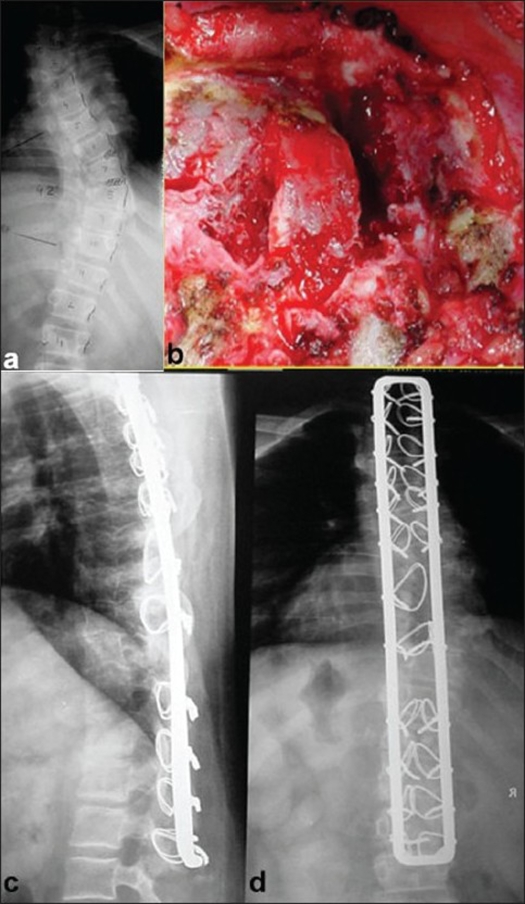
X-ray (Anteroposterior) of dorsolumbar spine (a) shows congenital scoliosis, (b) shows anterior epiphsiodesis. Postoperative X-ray lateral, (c) and Anteroposterior, (d) view of the same patient shows good correction and balance in sagittal and coronal planes

We observed average percentage Coronal Cobb's angle correction of 64.34 percent in the entire group and 69.19% in idiopathic group [Figures 3 and 4]. The best average percentage correction was achieved in the neuro muscular group at 73 % and the least in the congenital group at 60.2 percent [Figure 5]. The percentage correction in the neurofibromatosis group was 63.4%, though stastically it was not significantly relevant.

We observed the average degree of Cobb's angle correction was 47.13° in the entire group and 46° in the idiopathic group. The loss of correction at 24 months follow-up was 2.2° and at final follow up (avg - 6.5 years) averaged 5.8° in 21 patients.

The pre-operative thoracic hypokyphosis in 13 patients, averaging 14.03°, had average postoperative correction of 15.1° (range 8°-22°). Thoracic hyperkyphosis in 3 patients averaging 71.3° had average postoperative correction of 22.6° (range 4°-32°). There was lumbar hypolordosis in 12 patients averaging 32° with postop correction of 9° (range 6°-14°), and lumbar hyperlordosis in two patients averaging 71° with 9° correction.

Surgical time for only posterior surgery ranged from two hours 15 minutes to three hours 15 minutes (average of two hours and 40 minutes) and average blood loss was 650cc.Surgical time for antero-posterior surgeries ranged from four hours 15 minutes to eight hours 30 minutes (average of five hours) and average blood loss was 1200cc.

The lower extent of posterior spinal arthrodesis was a L1 (n= 2), L2 (n= 7) patients, L3 (n= 11) patients, L4 (n= 9) patients, L5 (n= 4) patients and S1 (n= 3).

The patients' satisfaction index as per the SRS24 questionnaire in 36 patients revealed that 80.2% of patients were ready to undergo the same surgery if required. On, whether they were happy to live with their present back status lifelong, 64.2% agreed. The pain scores improved marginally from 4.2 to 4.5 at 2 year follow up in 24 patients. Self image, how others perceived them after the surgery and satisfaction showed significant improvement after the procedure at a reasonable length of follow-up [Table 1].

**Table 1:** Patient satisfaction (SRS 24)

Post op (36)	Better (%)	Same (%)	Worse (%)
How others perceived them	67.5	30	2.5
Self image	63.9	27.8	8.3
Satisfaction	76.2	18.3	5.5

### Complications

**Figure 6:**
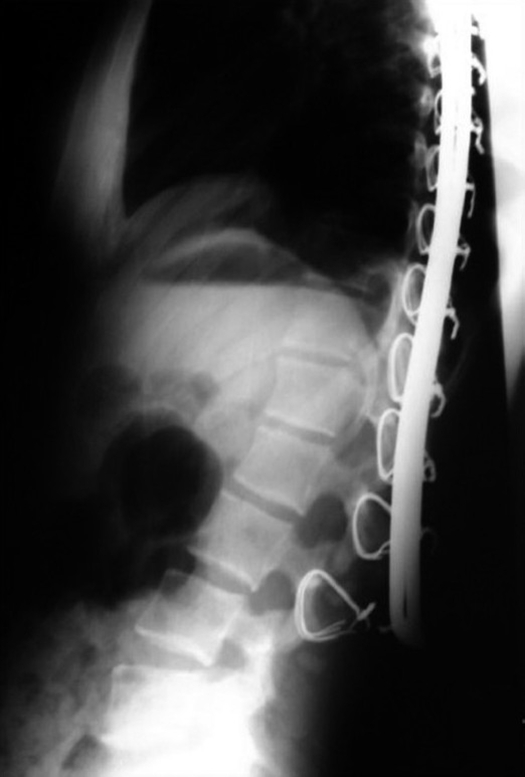
Lateral X-ray of dorsolumbar spine shows wire cut outs due to undercontouring of Hartshill rectangle

No neurological complications were seen in our series. Transient hyperasthesias were seen in two patients who were self limiting. None of the patients had loop rectangle (implant breakage, though 6 patients had breakage of sublaminar wires at the lower end of the instrumentation [Figure 6].

Asymptomatic implant prominence at lower end was seen in two patients, one of them warranted refashioning at a later date. One patient showed signs of decompensation after 1½ year follow-up, probably due to the inadequate lower extend of the arthrodesis.

In our study, the incidence of proximal junctional kyphosis was 19.4% of patients (7 of 36 patients), in whom PJK was noticed. The average angle was 14.2° till 2½ years postoperative follow up. The maximum noted value at last follow up was 21° in a neurofibromatosis patients 21 pts evaluated after 2½ years follow-up showed less progression thereafter. The upper extent of the instrumentation did not influence the incidence of PJK. Preoperative thoracic hyperkyphosis does show preponderance to PJK, since all the three patients with hyperkyphosis had maximum PJK values. However, it has to be emphasized here that no patient had any apparent clinically overt deformity due to the PJK.

No infections were seen in our series. Keloid formation was seen in five patients. Pseudarthroses were not seen in any patient till last follow-up.

## DISCUSSION

Treatment of scoliosis has made significant development in techniques and more so in the modalities of internal fixation. Most recent literature still popularizes the use of the third generation implants (Cotrel Dubousset etc;).^7^,^8^ Inadequate corrective forces in all planes was one reason for the sublaminar wires and loop rectangle implants to fall out of favor.^9^

An even distribution in corrective forces, with two lateral fixation points on each segments provide a good rotatory control as well. Sagittal plane correction is comparable to the third generation implants, as long meticulous care is taken in contouring the loop rectangle before tightening the wires onto it.^10^,^11^

We believe, especially so, in the idiopathic group It is the flexibility of the curve (the inherent character) rather than the type of instrumentation that determines the correctability.^12^ Moreover, sub laminar wires allow segmental fixation that requires only one-tenth of implanted metal in relation to hooks and screws. Thus larger bed surface area for the bone graft is available.

**Figure 4:**
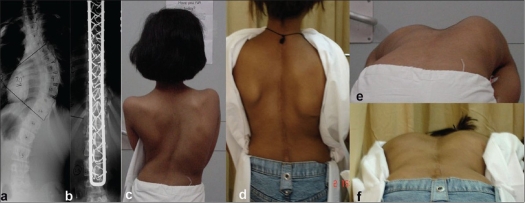
(a,b) Preoperative and post operative dorso lumbar x-rays in a girl with Turner's syndrome shows a good correction of scoliotic deformity. Clinical photographs (c-f) showing good correction and maintenance, alongwith rib hump improvement at 3 ½ years follow up

The deficiencies of possible deformation of implant due to long segment correction, in the originally used 5mm spinal loop rectangle, is nullified with the use of a sturdier 6 mm rod. Though rod rotation techniques may claim better corrective forces, overall spinal balance is appreciably achieved with the translation techniques as in the sub laminar wires. We also achieved significant de rotation, in the few cases we studied as seen in the Figure 4.

Sagittal reconstruction was highly dependent on the precontouring of the loop rectangle [Figure 5] and under contouring could lead to lower end cut of the sub laminar wires; it's a technical point to be well considered during surgery [Figure 6].

However, the extent of derotation achieved is not comparable to the screw – rod constructs. The loop rectangle and wire constructs aim at translation and coronal and sagittal balancing rather than derotation as its principle.

Sacral fixation with wires may be cumbersome and inadequate reason for which it may not be the implant of choice for pure lumbar curves. The loop rectangle and the wires also use the Harrington s stable zone principle of scoliosis correction which may call for longer constructs and the lower extent of arthrodesis may be longer as compared to the screw- rod constructs, though we are not sure how much it alters the end clinical functional result

The long term effects of sub laminar wires, including neurological complications, have been over-emphasized in literature. In their experience of more than 10 years with sub laminar, stainless steel wires, Plaza *et al*,^5^ generate the development of layers of fibrous tissue that isolate metal from dura. This acts like a protective shield, when occasionally the wires have to be removed and as a barrier to prevent spread of infection from bone or soft tissue to menninges. There has been no incidence of stenosis due to this fibrous tissue layers in our experience. As regards the neurological complications, we did not encounter any grave ones even in congenital deformities, though it must be stated here that MRI done for all, pre operatively, revealed no cord abnormalities. Thus the wire related adverse effects are comparable to any other system and is an aftermath of improper technique.^10^,^11^,^13^,^18^

Stability of the construct is comparable to any other system and there is no need of postoperative bracing in most patients.^7^–^9^,^12^,^14^ The risk of proximal junctional kyphosis is comparable to those in literature using other instrumentation techniques.^14^ It has, however, been argued that the longer constructs of loop rectangle and wires is automatically at a lesser risk for PJK as compared to the other constructs which aim at saving motion segments.

The locally made Hartshill rectangle and sublaminar wires are 3-4 times cheaper from locally made stainless steel and screw rod system and 10 times from imported titanium screw rod system.

A few concerns to be kept in mind while using the sub laminar wires and loop rectangle for scoliosis correction are - not to over tighten too much at each stage since it could end in wire cut outs – (tightening stage wise and sequentially makes the wire pretension itself at each step), upward pull at the wires while tightening to prevent looping underneath the lamina prevents dural or root damage.

Review of relevant recent literature of long term follow-up of different third generation implants in adolescent idiopathic scoliosis shows that our series have comparable results with respect to correction and maintenance of Cobb's angle and sagittal balance at long term follow ups though as expected our derotation were less and levels of fusion and instrumentation more.^15^–^17^

Benli IT *et al*,^15^ reported a minimum 10 year follow-up of adolescent idiopathic scoliosis treated with the TSRH system with average percentage Cobb's angle correction of 68.5 percent and loss of correction at last follow-up at around 8 – 10°.

In their series, Mueller *et al.*^16^ reported 62.5% correction in their series with an eight to 12 year follow-up. Remes *et al*.^17^ compared a long term follow-up of the CD and USS systems and reported similar correction percentages at 64–67% though they had significantly lower levels of instrumentation (9.8 levels) in comparison to our series (12.4 levels) which is a drawback with the loop rectangle and sub laminar wire systems. The clinical implications are, however, debatable.

## CONCLUSION

Significant correctability and ability to maintain correction in scoliotic spinal deformities in all planes, at a comparatively low cost, is a hall mark of segmental spinal fixation by locally made stainless steel Hartshill loop rectangle and sub laminar wires. Stability of the construct is excellent and the complications are over emphasized in literature. Appropriate technique is the key. This methodology and implantation technique is an asset for scoliotic deformity correction.
